# Bacterial translocation occurs early in cirrhosis and triggers a selective inflammatory response

**DOI:** 10.1007/s12072-023-10496-y

**Published:** 2023-03-07

**Authors:** Benedikt Simbrunner, Esther Caparrós, Teresa Neuwirth, Philipp Schwabl, Philipp Königshofer, David Bauer, Rodrig Marculescu, Michael Trauner, Bernhard Scheiner, Georg Stary, Mattias Mandorfer, Thomas Reiberger, Rubén Francés

**Affiliations:** 1 Division of Gastroenterology and Hepatology, Department of Medicine III, Medical University of Vienna, Vienna, Austria; 2 Vienna Hepatic Hemodynamic Laboratory, Medical University of Vienna, Vienna, Austria; 3 Christian Doppler Laboratory for Portal Hypertension and Liver Fibrosis, Medical University of Vienna, Vienna, Austria; 4 Ludwig Boltzmann Institute for Rare and Undiagnosed Diseases (LBI-RUD), Vienna, Austria; 5 CeMM Research Center for Molecular Medicine of the Austrian Academy of Sciences, Vienna, Austria; 6 CIBEREHD, Instituto de Salud Carlos III, Madrid, Spain; 7 Hepatic and Intestinal Immunobiology Group, Department of Clinical Medicine, Miguel Hernández University, San Juan de Alicante, Elche, Spain; 8 Instituto IDIBE, Miguel Hernández University, Elche, Spain; 9 Department of Dermatology, Medical University of Vienna, Vienna, Austria; 10 Department of Laboratory Medicine, Medical University of Vienna, Vienna, Austria; 11 Instituto ISABIAL, Hospital General Universitario de Alicante, Alicante, Spain

**Keywords:** Cirrhosis, Portal hypertension, Inflammation, Bacterial translocation, Gut–liver axis, Endotoxin, PAMPs, Circulatory dysfunction, Cytokine, Immunity

## Abstract

**Background:**

Experimental data suggest that bacterial translocation (BT) promotes systemic inflammation, portal hypertension, and circulatory dysfunction in advanced chronic liver disease (ACLD).

**Methods:**

Patients with ACLD undergoing hepatic venous pressure gradient (HVPG) measurement and absence of acute decompensation or infections were included (*n* = 249). Serum biomarkers of BT (lipopolysaccharide [LPS], lipoteichoic acid [LTA], bacterial DNA [bactDNA]), systemic inflammation and markers of circulatory dysfunction were assessed. T-cell subsets in intestinal biopsies (*n* = 7 ACLD, *n* = 4 controls) were analyzed by flow cytometry.

**Results:**

Patients had a median HVPG of 18 (12–21) mmHg and 56% had decompensated ACLD. LPS (0.04 [0.02–0.06] vs. 0.64 [0.30–1.06] EU/mL), LTA (4.53 [3.58–5.97] vs. 43.2 [23.2–109] pg/mL), and detection of bactDNA (≥ 5 pg/mL; 5% vs. 41%) were markedly higher in patients with ACLD than healthy controls (*n* = 40; *p* < 0.001) but were similar between different clinical stages of compensated and decompensated ACLD and displayed no meaningful correlation with HVPG and systemic hemodynamics. TNF-α and IL-10 correlated with LPS (Spearman’s *r*_s_ = 0.523, *p* < 0.001/*r*_s_ = 0.143, *p* = 0.024) but not with LTA. Presence of bactDNA was associated with higher LPS (0.54 [0.28–0.95] vs. 0.88 [0.32–1.31] EU/mL, *p* = 0.001) and TNF-α (15.3 [6.31–28.1] vs. 20.9 [13.8–32.9] pg/mL). Patients with ACLD exhibited a decreased CD4:CD8-ratio and increased T_H_1-cells in the intestinal mucosa as compared to controls. During a median FU of 14.7 (8.20–26.5) months, bacterial antigens did not predict decompensation or liver-related death (in contrast to HVPG, IL-6, and MAP) as well as infections at 24 months.

**Conclusion:**

BT occurs already in early ACLD stages and triggers a systemic inflammatory response via TNF-α and IL-10. Interestingly, BT markers showed no clear correlation with portal hypertension and circulatory dysfunction in patients with stable ACLD.

**Clinical trial number:**

NCT03267615.

**Supplementary Information:**

The online version contains supplementary material available at 10.1007/s12072-023-10496-y.

## Introduction

Recent research endeavors in the field of cirrhosis have indicated that bacterial translocation (BT) from the intestines plays a decisive role in advanced chronic liver disease (ACLD) [1]. Gut-derived bacteria and their products, i.e. pathogen-associated molecular patterns (PAMPs) may subsequently induce an immune response in the liver and other organs and promote systemic inflammation, portal hypertension, and circulatory dysfunction as key pathophysiological mechanisms in cirrhosis [1, 2].

Considering that BT requires disruption of multiple defense mechanisms [3], experimental animal studies suggested that BT in cirrhosis is related to dysbiosis, impaired antimicrobial peptide secretion, reduced mucus thickness, and downregulation of tight junction protein expression in the intestinal epithelium [4–6]. Concordantly, detection of bacterial DNA in mesenteric lymph nodes, blood and ascitic fluid of rats with cirrhosis was linked to serum levels of inflammatory cytokines [7]. Consequently, BT promotes a proinflammatory phenotype that is (at least partially) responsible for the association between systemic inflammation and liver-related complications in ACLD [2].

The onset of BT in cirrhosis was traditionally considered to parallel with the development of ascites, as suggested in early experimental studies [8, 9]. Accordingly, previous studies in humans investigating the link between circulating PAMPs and systemic inflammation and circulatory dysfunction have mostly focused on patients with ascites. For example, bacterial DNA in the blood of patients with ascites correlated with markers of systemic inflammatory response (e.g., tumor necrosis factor-alpha [TNF-α]) and endothelial or circulatory dysfunction [10]. However, other experimental and clinical studies found that bacterial antigens were already detectable in non-cirrhotic liver disease and compensated cirrhosis [11, 12]. Therefore, the presence and impact of BT across compensated and decompensated ACLD stages remain poorly characterized, particularly in stable patients without acute decompensation.

This study aimed to assess the link between circulating PAMPs and systemic inflammation, circulatory dysfunction, portal hypertension and clinical disease stages in a large cohort of consecutively recruited patients with stable ACLD undergoing liver vein catheterization. Furthermore, we analyzed the association between BT markers and disease progression and infections during follow-up.

## Patients and methods

### Study design, patient selection and clinical characterization.

Patients underwent hepatic venous pressure gradient (HVPG) measurement between 01/2017 and 08/2020 at the Vienna Hepatic Hemodynamic Lab, Medical University of Vienna, Austria, and were prospectively recruited in the Vienna Cirrhosis Study (VICIS). Portal hypertension (PH) and, thus, presence of ACLD was defined by an HVPG ≥ 6 mmHg. Patients with liver transplantation, pre/posthepatic/non-cirrhotic PH, transjugular intrahepatic portosystemic shunt, active extrahepatic malignant diseases, hepatocellular carcinoma out-of-Milan, non-selective betablockers, bacterial infection or non-elective hospitalization were excluded, resulting in a study cohort of 249 patients with stable ACLD (Supplementary fig. S1). Clinical disease stages were determined adapted to D’Amico et al. [13] and guidelines by the European Association for the Study of the Liver (EASL) [14]: stages (S) were defined as subclinical PH (S0; HVPG 6–9 mmHg), clinically significant PH (CSPH; S1-2; HVPG ≥ 10 mmHg without varices or presence of varices), previous variceal bleeding (S3), one non-bleeding decompensation event (S4), and ≥ 2 decompensation events (S5). Forty sex- and age-matched healthy individuals served as a control group for assessment of bacterial antigens in the systemic circulation.

### Measurement of hepatic venous pressure gradient and systemic hemodynamics

HVPG was assessed by liver vein catheterization in accordance with a standard operating procedure, as published previously [15]. Distinct steps of the procedure are outlined in the Supplementary material. Heart rate (HR) and non-invasive systolic, diastolic, and mean arterial pressure (MAP) were measured within the same session.

### Biomarker measurements

Biomarkers of BT and systemic inflammation (all patients), as well as circulatory dysfunction (i.e. renin and copeptin; available in 218 patients) were measured in serum and plasma obtained through the catheter introducer sheath during HVPG measurement. Personnel performing biomarker measurements was blinded to patient characteristics. C-reactive protein (CRP), interleukin-6 (IL-6), procalcitonin (PCT), lipopolysaccharide-binding protein (LBP), renin and copeptin levels were measured by the ISO-certified Department of Laboratory Medicine, Medical University of Vienna, following the manufacturers’ instructions. TNF-α and IL-10 were measured with commercially available ELISA kits (Human TNF-alpha and IL-10 Quantikine ELISA Kits from R&D Systems, Minneapolis, MN) with a detection limit of 6.23 pg/mL and 3.9 pg/mL, respectively, according to the manufacturer’s instructions. Lipopolysaccharide (LPS) levels were quantified using a quantitative chromogenic limulus amebocyte lysate (LAL) test (BioWhittaker, Nottingham, UK). Lipoteichoic acid (LTA) levels were assessed by Human LTA ELISA kit (Abbexa Ltd., Cambridge, UK). Bacterial DNA (bactDNA) was determined by broad-range polymerase chain reaction (PCR) of the 16S rRNA gene according to the methodology described elsewhere [16]. The presence of bactDNA was defined by a concentration of ≥ 5 pg/mL, while LPS and LTA detection limits were set at 0.25 UE/mL and 2.5 pg/mL, respectively [12]. Further details are outlined in the Supplementary material.

### Characterization of T-cell subsets in intestinal mucosa biopsies

Biopsies from the small intestine (duodenum) were obtained in seven patients with ACLD and four liver-healthy individuals undergoing endoscopy of the upper gastrointestinal tract with a standard biopsy forceps (Boston Scientific, MA, USA). T-cell subsets in the intestinal mucosa were characterized by multi-color flow cytometry analysis. Briefly, samples were digested, dissociated, and stained for multi-color flow cytometry to identify different T-cell subsets (αβT, γδT, CD8, CD4, T_H_1, T_H_2, T_H_17, Treg). More detailed information on sample processing and analysis is presented in the Supplementary material.

### Statistical analysis

Statistical analyses were performed using IBM SPSS Statistics 27 (IBM, Armonk, NY, USA) or GraphPad Prism 9 (GraphPad Software, La Jolla, CA, USA). Standard statistical methods were applied for descriptive statistics and comparison of categorical and continuous variables, as depicted in the Supplementary material. Correlation between continuous variables was determined by Spearman correlation coefficients (95% confidence interval). Kaplan–Meier curves with log-rank test as well as Cox proportional hazard models were used to assess determinants for the composite endpoint of first/further decompensation (incidence/worsening of ascites or hepatic encephalopathy, or development of variceal bleeding) or liver-related death, as well as the incidence of infections, at 24 months of follow-up. Patients were censored at the end of follow-up or liver transplantation. A two-sided *p* value < 0.05 denoted statistical significance for all analyses. Further information on statistical analyses is provided in the Supplementary material.

### Compliance with ethical standards

The study was conducted in accordance with the principles of the Declaration of Helsinki and its amendments, was approved by the local ethics committee of the Medical University of Vienna (EK1262/2017) and registered at clinicaltrials.org (NCT03267615). All patients provided written informed consent for liver vein catheterization and participation in the VICIS study. Patients and liver-healthy individuals undergoing endoscopy and duodenum biopsy gave written informed consent for endoscopic procedures and participation in the VICIS study and the VICIS control group, respectively. Healthy controls for measurement of bacterial antigens were recruited from the TraffEC study that was approved by the Ethics Committee of the Hospital General Universitario de Alicante, Spain.

## Results

### Patient characteristics

The study cohort of 249 patients had a median age of 59 (50–67) years, median HVPG of 18 (12–21) mmHg, median MELD of 11 (9–14) points, and the majority had male sex (*n* = 163, 65%). At the timepoint of HVPG measurement, 110 (44%) had compensated ACLD (cACLD), with 24 (9%) patients in S0 and 86 (34%) in S1-2. Furthermore, 139 (56%) patients had decompensated ACLD (dACLD), and 12 (5%) were in S3, 68 (27%) in S4, and 59 (24%) in S5 (Table 1; Supplementary table-S1). From patients classified as S5, 12 had refractory ascites, 6 had ascites and hepatic encephalopathy (HE) as well as history of variceal bleeding, 35 had ascites and HE, and 6 patients had history of variceal bleeding in combination with either ascites or HE. Twenty (8%) patients in the study cohort reported the intake of antibiotic prophylaxis at inclusion: 15 reported rifaximin intake for HE treatment, 4 patients norfloxacin for secondary spontaneous bacterial peritonitis (SBP) prophylaxis (SBP > 3 months prior to HVPG measurement), and 1 patient cotrimoxazole for pneumocystis prophylaxis.

**Table 1:** Patient characteristics across advanced chronic liver disease stages

	Compensated ACLD (*n* = 110)		Decompensated ACLD (*n* = 139)			*p* value
	Stage 0 (*n* = 24)	Stage 1–2 (*n* = 86)	Stage 3 (*n* = 12)	Stage 4 (*n* = 68)	Stage 5 (*n* = 59)	
Definition	HVPG 6–9	CSPH	Bleeding	Non-bleeding decompensation	Further decompensation	
Age (years)	53 (45–65)	60 (53–69)	60 (52–67)	59 (50–66)	58 (49–65)	0.572
Sex (M, %)	19 (79)	52 (61)	10 (83)	41 (60)	41 (70)	0.209
Etiology (*n*, %)						** < 0.001**
ALD	5 (21)	21 (24)	6 (50)	44 (64)	37(63)	
Viral	6 (25)	29 (34)	2 (17)	2 (3)	8(14)	
ALD + Viral	2 (8)	3 (4)	0 (0)	3 (4)	5(9)	
NASH	3 (13)	18 (21)	0 (0)	3 (4)	1(2)	
Cholestatic	0 (0)	5 (6)	0 (0)	4 (6)	1(0)	
Other	8 (33)	10 (11)	4 (33)	12 (18)	8 (14)	
HVPG (mmHg)	7 (6–8)	15 (12–19)	17 (13–19)	20 (15–22)	20 (17–24)	** < 0.001**
MELD Score (points)	8 (7–11)	10 (8–12)	10 (9–12)	12 (10–14)	13 (10–16)	** < 0.001**
HR (bpm)	78 (73–83)	75 (67–87)	70 (63–75)	80 (69–92)	72 (66–89)	0.152
MAP (mmHg)	109 (97–117)	106 (97–113)	106 (98–118)	98 (87–109)	97 (88–104)	** < 0.001**
HR/MAP ratio	0.75 (0.67–0.83)	0.73 (0.60–0.89)	0.66 (0.58–0.79)	0.79 (0.70–0.91)	0.78 (0.67–0.92)	**0.021**
Detectable bacterial antigens (*n*, %)						0.641
None	0 (0)	2 (2)	1 (8)	1 (2)	5(9)	
1	7 (29)	23 (27)	3 (25)	15 (22)	13(22)	
2	12 (50)	43 (50)	6 (50)	31 (46)	27(46)	
3	5 (21)	18 (21)	2 (17)	21 (31)	14 (24)	
LPS (EU/mL)	0.96 (0.47–1.28)	0.68 (0.31–1.03)	0.43 (0.19–1.16)	0.70 (0.31–1.15)	0.57 (0.21–1.02)	0.347
LTA (pg/mL)	36.8 (23.7–190)	34.5 (21.0–76.8)	39.8 (30.3–114)	51.8 (26.1–109)	48.3 (23.6–113)	0.324
BactDNA (n, %)	8 (33)	38 (44)	2 (17)	30 (44)	23 (39)	0.373
WBC (G/L)	5.53 (3.87–6.83)	4.74 (3.31–6.02)	3.12 (2.39–5.13)	4.69 (3.29–5.94)	3.99 (3.17–5.46)	**0.047**
CRP (mg/dL)	0.14 (0.06–0.29)	0.20 (0.09–0.39)	0.15 (0.09–0.26)	0.36 (0.14–0.74)	0.37 (0.15–0.75)	** < 0.001**
IL-6 (pg/mL)	4.25 (2.76–8.26)	5.56 (3.43–8.74)	5.48 (3.38–7.64)	8.40 (5.26–12.6)	10.8 (6.90–22.8)	** < 0.001**
IL-10 (pg/mL)	13.2 (9.05–18.7)	13.3 (9.95–18.0)	14.0 (11.9–17.5)	11.6 (8.98–15.5)	11.4 (8.70–14.7)	0.152
TNF-α (pg/mL)	22.9 (14.2–34.2)	19.9 (12.9–31.5)	29.6 (6.79–32.5)	16.0 (10.7–24.7)	15.7 (8.10–25.4)	0.080
Procalcitonin (ng/mL)	0.04 (0.03–0.07)	0.07 (0.05–0.11)	0.05 (0.04–0.09)	0.10 (0.05–0.15)	0.11 (0.06–0.16)	** < 0.001**
LBP (µg/mL)	7.30 (5.73–9.53)	6.55 (5.38–8.40)	7.30 (5.52–9.35)	6.96 (4.66–8.32)	6.39 (4.92–8.33)	0.559
Copeptin (pmol/L)	8.90 (4.66–20.7)	5.73 (3.28–13.2)	7.24 (4.36–10.8)	9.20 (4.91–17.5)	11.1 (5.17–16.7)	0.073
Renin (µIU/mL)	17 (8.80–29.5)	11.3 (4.88–33.3)	10.0 (3.83–38.6)	55.5 (15.2–177)	115 (30.2–354)	** < 0.001**
Antibiotic prophylaxis (n, %)	1 (4)	0 (0)	0 (0)	2 (3)	17 (29)	** < 0.001**

*p*-values < 0.05 are indicated in bold

*ALD* alcohol-related liver disease, *bactDNA* bacterial DNA, *CRP* C-reactive protein, *HR* heart rate, *HVPG* hepatic venous pressure gradient, *IL-6/-10* interleukin-6/-10, *LBP* lipopolysaccharide binding protein, *M* male sex, *MAP* mean arterial pressure, *MELD* Model of End Stage Liver Disease, *NASH* non-alcoholic steatohepatitis, *PCT* procalcitonin, *TNF-α* tumor necrosis factor-alpha, *WBC* white blood cell

### Bacterial translocation across disease stages of advanced chronic liver disease

**Fig. 1:**
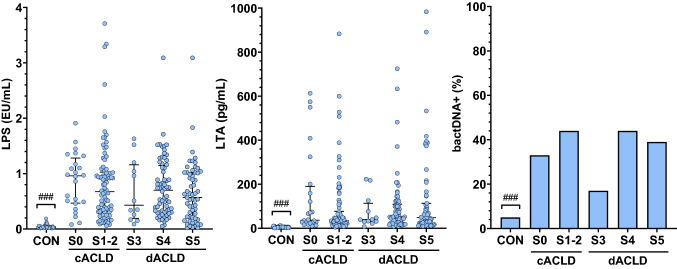
Bacterial antigens in the systemic circulation across clinical advanced chronic liver disease (ACLD) stages. Statistical analysis: Mann–Whitney *U* test was applied to compare continous variables in patients stratified by the presence of bactDNA. Chi-squared test was used to compare the presence of bactDNA between clinical disease stages. Legend: (###) *p* < 0.001 vs. patients with ACLD. *c/dACLD* compensated/decompensated advanced chronic liver disease, *LTA* lipoteichoic acid, *LPS* lipopolysaccharide, *bactDNA* bacterial DNA

Patients displayed significantly elevated levels of bacterial antigens as compared to healthy controls: median LPS concentration was 0.64 (0.30–1.06) EU/mL (vs. 0.04 [0.02–0.06] EU/mL in controls; *p* < 0.001), median LTA 43.2 (23.2–109) pg/mL (vs. 4.53 [3.58–5.97] EU/mL in controls; *p* < 0.001), and bactDNA was detected in 101 (41%) patients (vs. 5% [*n* = 2] in controls; *p* < 0.001). Circulating LPS, LTA, and bactDNA were not related to disease stage, indicating that BT already occurs early in cACLD and that the amount of circulating bacterial antigens is not clearly contingent on disease severity (all *p* > 0.05; Fig. 1). None of the evaluated antigens could be detected in 9 (4%) patients, whereas 1 antigen was detected in 61 (24%), 2 antigens in 119 (48%), and 3 antigens in 60 (24%) patients (Supplementary Table S1). Detection of bactDNA was related to a significant increase of LPS (0.88 [0.32–1.31] vs. 0.53 [0.28–0.95] EU/mL in patients without bactDNA, *p* = 0.001) but not LTA (43.2 [24.7–123] vs. 43.4 [22.1–99.9] pg/mL in patients without bactDNA, *p* = 0.471). Furthermore, LPS levels did not correlate with LTA in our cohort (*p* = 0.793; Supplementary Fig. S2). Bacterial antigen levels were also statistically similar when comparing patients stratified by cACLD and dACLD (S0-S2 vs. S3-S5; all *p* > 0.05; Supplementary Table S2), ascites grading (Supplementary Fig. S3), patients stratified by etiology (*p* > 0.05; Supplementary Fig. S4), or when excluding patients on prophylactic antibiotic medication (all *p* > 0.05; Supplementary Fig. S5) in further exploratory analyses.

### The link between systemic inflammation and circulating bacterial antigens

**Fig. 2:**
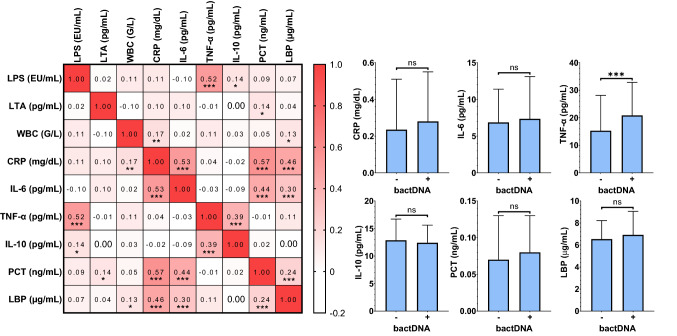
Relationship between bacterial antigens and inflammation markers in the systemic circulation. Statistical analysis: Spearman’s correlation coefficients were calculated to assess the association between continous variables. Mann–Whitney *U* test was applied to compare continous variables. *ns* not significant, (*) *p* < 0.05, (**) *p* < 0.01, (***) *p* < 0.001. *LTA* lipoteichoic acid, *LPS* lipopolysaccharide, *bactDNA* bacterial DNA, *TNF-α* tumor necrosis factor-alpha

Since BT is considered to induce a systemic inflammatory response, we investigated the relation between bacterial antigens and inflammation biomarkers. In line with a previous study from our center [17], systemic inflammation markers CRP, IL-6, and PCT increased across disease stages. Conversely, TNF-α, IL-10, and LBP levels were not associated with disease severity (Table 1). TNF-α levels correlated with LPS (*r*_s_ = 0.523, 0.42–0.61, *p* < 0.001) and increased significantly in patients with detectable bactDNA (20.9 [13.8–32.9] vs. 15.3 [9.31–28.1] in patients without bactDNA, *p* < 0.001), however, TNF-α levels did not correlate with LTA (*p* = 0.869; Fig. 2). Furthermore, LPS exhibited a weak correlation with IL-10 (*r*_s_ = 0.143, 0.02–0.27, *p* = 0.024). All other inflammatory biomarkers investigated in the present study exhibited a largely consistent correlation, however, did not exhibit a meaningful correlation to bacterial antigen levels. Interestingly, LBP levels showed no association with LPS, LTA, and bactDNA (Fig. 2; Supplementary Figs. S6/S7). TNF-α correlated significantly with the anti-inflammatory cytokine IL-10 (*r*_s_ = 0.395, 0.28–0.50, *p* < 0.001) but was not directly associated with CRP, IL-6, procalcitonin or LBP (all *p* > 0.05; Fig. 2).

### Hepatic and systemic hemodynamics and their relation with bacterial translocation

**Fig. 3:**
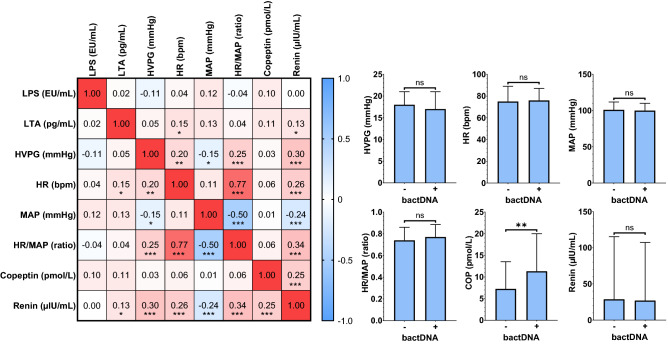
Relationship between bacterial antigens and measures of hepatic and systemic hemodynamics. Statistical analysis: Spearman’s correlation coefficients were calculated to assess the association between continous variables. Mann–Whitney *U* test was applied to compare continous variables. *ns* not significant, (*) *p* < 0.05, (**) *p* < 0.01, (***) *p* < 0.001. *LTA* lipoteichoic acid, *LPS* lipopolysaccharide, *bactDNA* bacterial DNA, *TNF-α* tumor necrosis factor-alpha

Next, we investigated whether HVPG and HR, MAP, and the HR/MAP ratio were linked to bacterial antigen levels. HVPG increased and MAP decreased across disease stages. Renin levels significantly increased across disease stages and exhibited a pronounced increase in S4 and S5 (*p* < 0.001), while copeptin tended to increase in S4 and S5 (*p* = 0.073; Table 1). HVPG correlated significantly with HR (*r*_s_ = 0.196, 0.07–0.32, *p* = 0.002), MAP (*r*_s_ =  − 0.146, − 0.27 to − 0.02 *p* = 0.024), HR/MAP ratio (*r*_s_ = 0.251, 0.13–0.37, *p* < 0.001), consolidating the link between PH severity and hyperdynamic circulation. From an overall perspective, BT markers showed no meaningful correlation with HVPG and other measures of systemic hemodynamics, however, copeptin levels were significantly higher in patients with detectable bactDNA and LTA showed a statistically significant but very weak correlation with renin levels (Fig. 3; Supplementary Fig. S8).

### Prediction of disease progression and infections by bacterial translocation markers

Based on the pathophysiological concept that BT promotes disease progression in ACLD, we assessed whether bacterial antigens were predictive for the composite endpoint of first/further decompensation or liver-related death. Furthermore, it was investigated whether bacterial antigens indicated the development of infections. The median follow-up period was 14.7 (8.20–26.5) months. Seven (3%) patients were lost to follow-up and not considered for the analysis. During the follow-up period, first/further decompensation events or liver-related deaths at 24 months were recorded in 66 (27%) patients of our study cohort. Furthermore, incidence of infection was recorded in 36 (15%) patients at 24 months: *n* = 9 patients developed respiratory infections, *n* = 7 SBP, *n* = 7 sepsis, *n* = 5 urinary tract infections, *n* = 2 gastrointestinal infections, *n* = 2 other causes (*n* = 1 secondary peritonitis; *n* = 1 bacterial vaginosis), and *n* = 4 had an unknown focus.

Kaplan–Meier curves were drawn in patients stratified by presence of bactDNA as well as median LPS and LTA levels, respectively. However, log-rank tests indicated no difference in decompensation or liver-related death (LPS: *p* = 0.520; LTA: *p* = 0.106; bactDNA: *p* = 0.273) and infections (LPS: *p* = 0.771; LTA: *p* = 0.428; bactDNA: *p* = 0.883; Supplementary Fig. S9).

Finally, bacterial antigens and other variables indicating disease severity or systemic inflammation were entered into a Cox proportional hazard model to determine predictors of decompensation or liver-related mortality. Bacterial antigens were, again, not predictive for this endpoint. In contrast, HVPG (aHR 1.08, 95%CI 1.03–1.12, *p* < 0.001), MAP (HR 0.97, 95%CI 0.96–0.99, *p* = 0.001), and IL-6 (HR 1.03, 95%CI 1.01–1.04, *p* < 0.001) exhibited independent prognostic value for this endpoint (Table 2).

**Table 2:** Cox proportional hazard regression model assessing predictors of first/further decompensation or liver-related death

Overall cohort (*n* = 249)	First/further decompensation or liver-related mortality during follow-up					
	Univariate analysis			Multivariate analysis		
Patient characteristics	HR	95%CI	*p* value	HR	95%CI	*p* value
Age (per year)	1.00	0.98–1.02	0.876			
MELD (per point)	1.10	1.04–1.17	** < 0.001**	1.05	0.97–1.13	0.235
HVPG (per mmHg)	1.09	1.05–1.14	** < 0.001**	1.08	1.03–1.12	** < 0.001**
MAP (per mmHg)	0.97	0.95–0.98	** < 0.001**	0.97	0.96–0.99	**0.001**
IL-6 (per pg/mL)	1.02	1.01–1.02	**0.002**	1.03	1.01–1.04	** < 0.001**
LPS (per EU/mL)	0.75	0.47–1.18	0.212			
LTA (per 10 pg/mL)	1.01	0.99–1.02	0.138			
BactDNA + (≥ 5 pg /mL)	1.31	0.81–2.14	0.275			
TNF-α (per pg/mL)	0.99	0.97–1.01	0.259			
IL-10 (per pg/mL)	0.99	0.94–1.04	0.625			

*p*-values < 0.05 are indicated in bold

*bactDNA* bacterial DNA, *HVPG* hepatic venous pressure gradient, *IL-6/-10* interleukin-6/-10, *MAP* mean arterial pressure, *MELD* Model of End Stage Liver Disease, *TNF-α* tumor necrosis factor-alpha

### T-cell profile in the intestinal mucosa of patients with ACLD

**Fig. 4:**
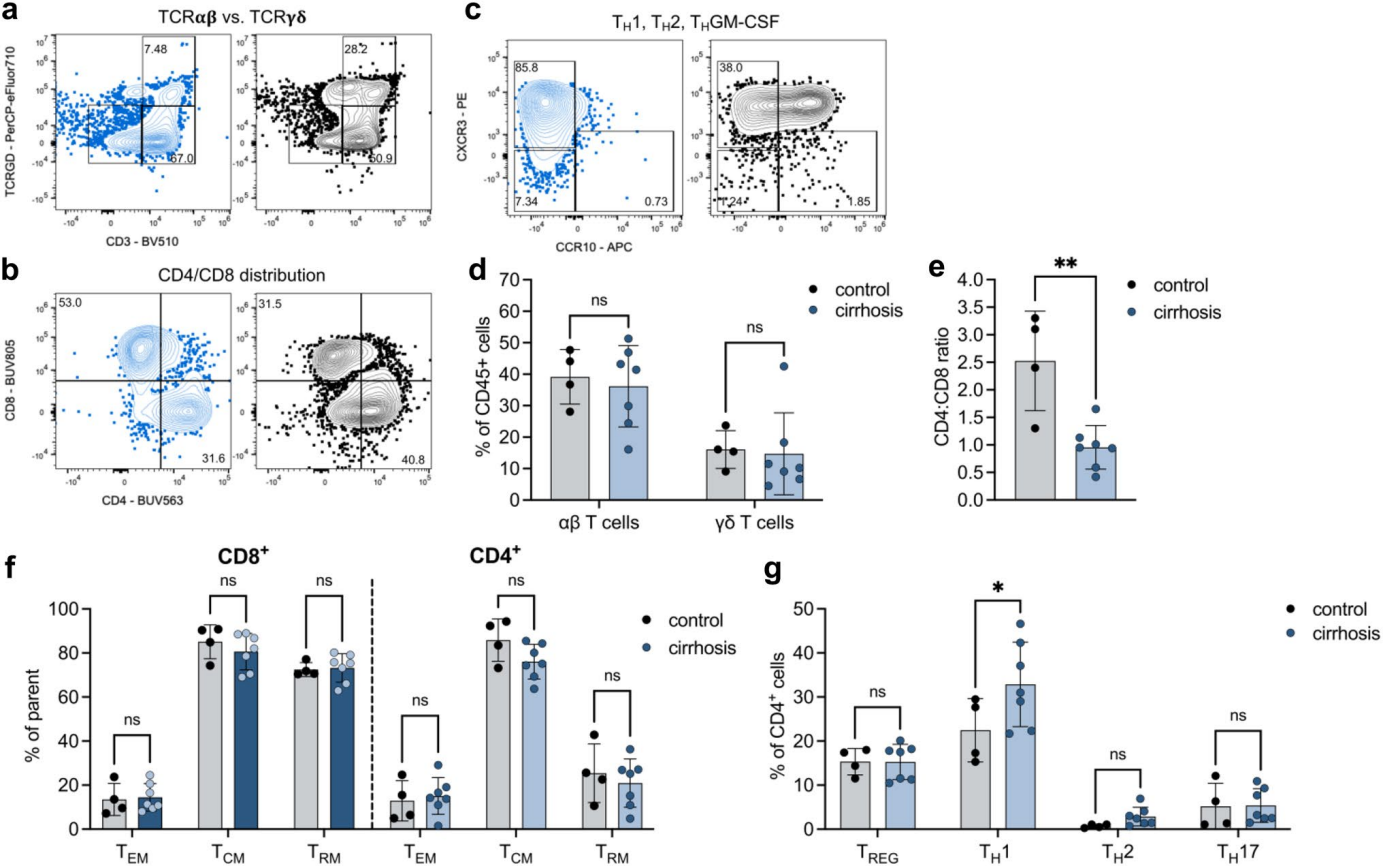
T-cell subsets in the intestinal mucosa from patients with cirrhosis. Legend and statistical analysis: **A**-**C** Representative FACS plots of the distribution of αβT and γδT cells (**A**), CD4 and CD8 T cells (**B**) and TH1/2 cells (**C**). **D** Quantification of αβT and γδT cells, 2-way ANOVA with multiple comparison, Sidak-adjusted. **E** CD4:CD8 ratio in patients vs. control, unpaired *t* test (**F**). Quantification of memory CD4 and CD8 T cells, 2-way ANOVA with multiple comparison, Sidak-adjusted. **G** Quantification of helper T cell subsets, 2-way ANOVA with multiple comparison, Sidak-adjusted. Patients *n* = 7, liver-healthy controls *n* = 4

To investigate whether the observation that bacterial antigens exhibited a significant difference between liver-healthy individuals and in patients with ACLD aligned with changes in the composition of immune cells in the intestinal mucosa, we assessed T-cell subsets in duodenum biopsies of patients with ACLD and liver-healthy controls (Fig. 4A–C; Supplementary Fig. S10). While no change in the distribution of αβT- and γδT-cells was observed between patients and controls (Fig. 4D), patients with cirrhosis showed a significant decrease in the CD4:CD8 T-cell ratio (Fig. 4E) reflecting a higher number of CD8 + T-cells in the intestinal mucosa as compared to controls. Furthermore, we observed an increase in T_H_1-cells and a non-significant increase in T_H_2-cells. Memory T-cell subsets (CD4 and CD8) remained unchanged, although there is a tendency towards a decrease in the CD4 + central memory T-cell pool (Fig. 4F). No changes were seen in regulatory T-cells (Treg) and T_H_17 cells (Fig. 4G).

## Discussion

The present study investigated the link between bacterial antigens in the systemic circulation and disease severity, portal hypertension, hemodynamic dysfunction, and systemic inflammation in a well-characterized cohort of 249 patients with different clinical stages of compensated and decompensated ACLD undergoing HVPG measurement. Notably, patients with acute hepatic decompensation or bacterial infections were excluded to determine the significance of circulating bacterial antigens in patients with a rather stable steady state of ACLD. The underlying hypothesis for the present study is based on the significance attributed to BT in ACLD, as it is considered to occur due to impairment of multiple intestinal defense mechanisms, thus, enabling the crossing of pathogens and PAMPs across the intestinal barrier into the portal venous system [1, 18]. Consequently, BT may promote systemic inflammation, portal hypertension, circulatory dysfunction, and thus, directly impact on disease progression [2, 3].

We found that bacterial antigens in the systemic circulation were markedly higher in patients with ACLD, as compared to a sex-/age-matched control group, which confirms the concept that BT is an important feature of ACLD. LPS levels in patients with ACLD were higher in the presence of bactDNA, indicating that certain PAMPs tend to occur simultaneously in the systemic circulation. The concurrent presence of different bacterial antigens was also reported by Gómez-Hurtado et al. in patients with non-alcoholic fatty liver disease (NAFLD), particularly in patients with advanced fibrosis [12]. The absence of a correlation between LTA (primarily a component of gram-positive bacteria) and LPS as well as bactDNA may also reflect the relatively more abundant colonization of gram-negative as compared to gram-positive bacteria in cirrhosis [19]. Interestingly, our study found that the concentrations or presence of bacterial antigens were statistically similar between patients with cACLD and dACLD (and the respective subgroups), suggesting that BT may already occur in early (compensated) stages of ACLD and exhibits no apparent dynamics across the spectrum of (d)ACLD. At the first glance, these results seem in conflict with the widely propagated concept that BT primarily occurs in dACLD [2], as suggested by animal studies that demonstrated the occurrence of BT in cirrhotic rats with ascites [8, 9, 20], but also earlier studies in humans that suggested an elevation of LPS levels in patients with ascites [21–23]. Of note, these and other previous studies had a considerably smaller sample size [21–23], and in the study by Albillos et al., the reported increase of LPS was only restricted to a subgroup of patients with ascites [22]. In our study, patients stratified by the presence or severity of ascites displayed similar bacterial antigen levels. Concordantly, a study by Genesca et al. also reported no association between LPS levels and presence of ascites or disease severity in patients with ACLD [24]. Similar to aforementioned studies, our study is limited by not reporting bacterial antigen levels from portal venous blood, thus, not being able to quantify hepatic clearance of PAMPs originating from BT in the intestines — which might be quite functional even in the setting of chronic liver disease [25]. Furthermore, we acknowledge that some subgroups of clinical disease stages had relatively small sample sizes, which may relate to limitations towards the robustness of the results in certain disease stages (e.g. in S3). Nevertheless, exploratory analyses on the comparison of cACLD and dACLD patients, and after exclusion of patients on prophylactic/poorly absorbable antibiotics, displayed the same results. Therefore, our results indicate that the concept of BT being an exclusive feature of dACLD (or patients with ascites) should be revisited.

Moreover, we investigated whether bacterial antigens were associated with systemic inflammation levels in patients with ACLD. BT is considered a major factor contributing to systemic inflammation [2], which commonly increases in patients with dACLD and holds an important prognostic value in patients with both stable and acutely decompensated ACLD [17, 26, 27]. The detrimental pathophysiological role of BT on the induction of hepatic and systemic inflammatory processes in the setting of liver cirrhosis has been documented by numerous experimental studies in animals [28–31]. LPS and the presence of bactDNA correlated significantly with the inflammatory cytokine TNF-α, which is in line with a previous study focusing on patients with ascites [32], and displayed a weak correlation with IL-10. Surprisingly, no meaningful correlation between bacterial antigens and other inflammation markers (e.g., CRP, IL-6, …) were observed in our study. The results suggest a selective systemic inflammatory response in the presence of PAMPs, but also indicate that commonly used systemic inflammation parameters such as CRP or IL-6, that have been linked to disease severity/progression and prognosis in multiple previous studies [17, 27, 33–35], do not necessarily reflect the—at least momentary—presence of BT antigens in patients with (rather stable) ACLD.

Two contributing factors help explain this. First, CRP and IL-6 are acute-phase proteins that are secreted by the liver in response to hepatic damage, independently of other inflammatory triggers such as bacterial antigens (therefore independent of them). In second place, BT episodes have been described as recurrent events during advanced chronic liver disease. A sequential study following cirrhotic patients every eight hours for three days revealed a highly dynamic clearance of bacterial DNA in blood [16] and suggested that transversal studies may be biased by the highly flexible rate and time interval of BT episodes. Furthermore, a compensatory tolerogenic response is also mounted to balance bacterial antigen-driven inflammation [36]. Nevertheless, we acknowledge that that our study may not capture certain confounding factors promoting BT or influencing the presence of bacterial antigens in the systemic circulation (including the use of non-/poorly absorbable antibiotics).

On the background that BT may promote portal hypertension and circulatory dysfunction in ACLD [3], our study addressed whether bacterial antigens were linked to hepatic and systemic hemodynamics. Experimental studies have demonstrated that BT is directly linked to the development of sinusoidal endothelial dysfunction and portal hypertension [37, 38], and also studies in humans have linked the detection of bacterial antigens or LBP to portal hypertension and systemic hemodynamic dysfunction [22, 39, 40]. Conversely, no clinically meaningful link between bacterial antigens and HVPG, MAP, HR/MAP ratio, or soluble markers of circulatory dysfunction were observed in our study. Considering the comparatively large sample size of the present study cohort, one may speculate that bacterial antigens are simply not well-suited to represent these measures in a relatively stable patient population. Concordantly, bacterial antigens were also not indicative of disease progression, in contrast to well-established measures such as HVPG, IL-6, or MAP. Furthermore, bacterial antigens did not predict the development of infections. In this context, a plausible hypothesis would establish that in patients with stable ACLD, BT challenges are better controlled, and antigen clearance mechanisms start a moderate systemic inflammatory response that in early stages do not correlate with the loss of hepatic function. During decompensation, the recurrent systemic inflammatory response further increases due to a significantly altered gut permeability, correlating with a deranged hepatic immune clearance and the general failure of liver functional activity [36].

Finally, we characterized T-cells in small intestinal biopsies of patients with ACLD and liver-healthy controls and found a relative abundance of CD8 T-cells (in relation to CD4 T-cells) and increased T_H_1 cells. Considering previous reports on changes in gut barrier integrity in the small intestine of patients with cirrhosis [41] and the link between TNF-α and the stimulation of T-cells [42], our results provide novel translational evidence on changes of the immune cell profile in the intestinal mucosa that may be related to BT. Therefore, further translational studies are needed to better understand immunological changes in the gut-liver axis and their link to the microbiota composition and BT in ACLD.

In summary, our study demonstrates that BT may already occur in compensated ACLD, and seems to trigger a selective inflammatory response, independent of hepatic damage progression. In fact, BT markers were not linked to disease stages and not suited to indicate portal hypertension, systemic inflammation, or circulatory dysfunction. Future studies need to investigate the longitudinal presence and dynamics of BT in patients with ACLD.

## Supplementary Information

Below is the link to the electronic supplementary material.

Supplementary file1 (PDF 2320 KB)

## Data Availability

Data are available upon reasonable request to the corresponding author.

## Abbreviations

bactDNA: Bacterial DNA
BT: Bacterial translocation
c/dACLD: Compensated/decompensated advanced chronic liver disease
CRP: C-reactive protein
CSPH: Clinically significant portal hypertension
EASL: European Association for the Study of the Liver
FACS: Fluorescence-activated cell sorting
HE: Hepatic encephalopathy
HR: Heart rate
HVPG: Hepatic venous pressure gradient
IL-6/-10: Interleukin-6/-10
LAL: Limulus amoebocyte lysate
LBP: Lipopolysaccharide-binding protein
LPS: Lipopolysaccharide
LTA: Lipoteichoic acid
MAP: Mean arterial pressure
MELD: Model of End Stage Liver Disease
NAFLD: Non-alcoholic fatty liver disease
PAMPs: Pathogen-associated molecular patterns
PCR: Polymerase chain reaction
PCT: Procalcitonin
PH: Portal hypertension
SBP: Spontaneous bacterial peritonitis
TNF-α: Tumor necrosis factor-alpha
