# Supplementary material for: Bacterial translocation occurs early in cirrhosis and triggers a selective inflammatory response
Source: Hepatol Int. 2023 Mar 7;17(4):1045–56. doi: 10.1007/s12072-023-10496-y (PMC10386924; doi:10.1007/s12072-023-10496-y)
Supplement: Supplementary file 1 — Supplementary file1 (PDF 2320 KB) [file 12072_2023_10496_MOESM1_ESM.pdf]

# SUPPLEMENTARY MATERIAL

**Title: Bacterial translocation occurs early in cirrhosis and triggers a selective inflammatory response**

## Table of contents

Supplementary methods .... 2

Measurement of hepatic venous pressure gradient .... 2

LPS measurement .... 2

Statistical analysis .... 2

Definition of liver-related events .... 3

Supplementary tables .... 4

Supplementary figures .... 10

Patient flow chart .... 10

Relationship between different bacterial translocation markers .... 11

Bacterial translocation markers and disease stage, ascites, or etiology .... 11

Bacterial translocation markers and systemic inflammation .... 14

References (used in the supplementary material) .... 19

## Supplementary methods

*Measurement of hepatic venous pressure gradient*

Measurement of hepatic venous pressure gradient (HVPG) were performed following a standard operating procedure in fasting condition [1]. Briefly, under local anaesthesia and ultrasound guidance, a catheter introducer sheath was placed in the right internal jugular vein. Subsequently, a balloon catheter was advanced into inserted into a large hepatic vein *via* the inferior vena cava under fluoroscopic guidance. Correct and sufficient wedge position of the catheter was ensured by injecting contrast media while the inflated balloon was obstructing the outflow of the cannulated hepatic vein. At least three measurements of free and wedged hepatic vein pressure were performed to assess HVPG.

*LPS measurement*

A quantitative chromogenic limulus amoebocyte lysate (LAL) test (BioWhittaker, Nottingham, UK) was followed to evaluate LPS levels. Due to LPS ubiquity, samples and reagents were handled in an airflow chamber and processed with pyrogen-free material tested by manufacturers. E. coli lyophilized endotoxin (22 UE/ml) provided by the kit was used to set standard endotoxin concentrations ranging from 5.0 UE/ml to 0.1 UE/ml. To verify the lack of product inhibition by plasma protein, a dilution/heating inactivation protocol was followed prior to endotoxin measurement. A pooled E. coli endotoxin spike solution (0.4 UE/ml) was prepared with serum samples. Dilutions ranging from 1/2 to 1/20 were performed over spiked and unspiked serum samples. All test samples were then incubated at 60°C during 30 min [2]. The LAL test was performed after this period. The non-inhibitory dilution was established when the difference between spiked and unspiked endotoxin values was equal to the known concentration of the spike ±25%, as detailed by the manufacturer. Final sample dilutions used were 1/10 (spike recovery after correction of dilution: 0.34 UE/ml). All samples were tested in triplicate and read at 405 nm in a Epoch 2 microplate reader (Agielnt, Santa Clara, CA).

*Statistical analysis*

Continuous variables are reported as mean ± standard error of the mean or median with interquartile range (IQR) and categorical variables are displayed as absolute (n) and relative (%) frequencies. Normal distribution was determined by Kolmogorov-Smirnov and Shapiro-Wilk tests. Continuous variables were compared by Student’s t test, Mann-Whitney U test, Kruskal-Wallis test, or Analysis of variance (ANOVA), as applicable. Post-hoc comparisons

were performed by Tukey's or Dunn's multiple comparisons tests. Categorical variables were compared by Chi squared or Fisher's Exact test.

*Definition of liver-related events*

The endpoint "first/further decompensation or liver-related death" was determined as the first occurrence and/or aggravation of variceal bleeding, ascites, hepatic encephalopathy (HE), or incidence of liver-related death within the follow-up period. The timepoint of HVPG measurement was defined as baseline, and the time to the closest event of interest was determined. Patients were censored at last clinical contact, transplantation, or at 24 months of no event occurred within 24 months of follow-up. The third large-volume paracentesis within 6 months was defined as worsening of ascites in patients without refractory ascites who had already received treatment for ascites at the timepoint of HVPG measurement. Admission for overt HE was defined as worsening of HE in patients who had already received HE treatment and/or mild HE but had no record of overt HE prior to HVPG measurement.

*Characterization of T-cell subsets in the intestinal mucosa*

<u>Patient characteristics</u>

Patients with ACLD undergoing endoscopy (n=7; identical in- and exclusion criteria) had a median HVPG of 11 (9-20) mmHg, a median MELD of 10 (9-13) points, and 5 (71%) patients had male sex. Four patients had alcohol-related liver disease (ALD), and 3 patients had non-alcoholic steatohepatitis (NASH). Liver-healthy individuals were slightly younger with a median age of 51 (33-58) years, while patients with ACLD had a median age of 59 (52-62) years.

<u>Tissue digestion</u>

Biopsies were placed in RPMI + 10% FBS and processed immediately. Biopsies were washed in PBS prior to digestion. The tissue was minced into 2x2mm pieces with a scalpel and digested for 2.5h with the Whole Skin Dissociation Kit (Miltenyi Biotec, cat.: 130-101-540) without Enzyme P to preserve surface epitopes for flow cytometry. The tissue was then dissociated using the gentleMACSTM Dissociator (Miltenyi Biotec, cat.: 130-093-235) using the h_tumor_02 program. Cell suspension was then passed through a 70uM filter, washed with 20mL 10% FBS in PBS, and spun down at 300g for 5min. Cells were washed with PBS and stained for flow cytometry.

Staining for multi-color flow cytometry

Cells were stained in 50uL staining mix, containing PBS and antibodies at respective concentrations (see Panel below). Cell suspension in the antibody mix was incubated at 4°C for 30min in the dark. Subsequently cells were washed with FACS buffer (1% BSA, 2mM EDTA in PBS). Samples were then resuspended in 200uL FACS buffer and kept on ice until acquisition.

Acquisition and analysis

Samples were acquired on Cytek® Aurora (Cytek®), using a 5-laser setup. Anti-Mouse Ig, κ/Negative Control Compensation Particles (BD, cat.: 552843) were used for single stains with mouse isotype antibodies and tissue-derived cells for non-mouse antibodies which were then used for unmixing. Fluorescence minus one (FMO) stains were performed as a control. $T_H$1-cells were defined by "CD4+CCR7+CD45RA-CXCR5-CCR6-CXCR3+CCR10-", $T_H$2-cells by "CD4+CCR7+CD45RA-CXCR5-CCR6-CXCR3-CCR10-", regulatory T-cells (Treg) by "CD4+CD127lo/-CD25+/hi", and $T_H$17 cells by "CD4+CCR7+CD45RA-CXCR5-CCR4+CCR6+CXCR3+CCR10-". Analysis was performed in FlowJo (v.10.8.1) and GraphPad Prism (v.9.4.1).

Panel

| Marker | Fluor | Clone | Supplier | Cat# | Isotype | Laser |
|---|---|---|---|---|---|---|
| Live/Dead | Zombie UV | n/a | Biolegend | 423107 | n/a | UV6 |
| CD45 | PerCP | HI30 | Biolegend | 304026 | Mouse IgG1, κ | B8 |
| CD3 | BV510 | SK7 | Biolegend | 344828 | Mouse IgG1, κ | V7 |
| CD4 | BUV563 | OKT4 | BD | 750979 | Mouse IgG1, κ | UV9 |
| CD8 | BUV805 | SK1 | BD | 612889 | Mouse BALB/c IgG1, κ | UV16 |
| CD45RA | BUV395 | 5H9 | BD | 740315 | Mouse IgG1, κ | UV2 |
| CCR7 | PE-CF594 | 150503 | BD | 562381 | Mouse IgG2a | YG3 |
| CD127 | APC-eF780 | RDR5 | Thermo Fisher | 47-1278-42 | Mouse IgG1, κ | R7 |
| CD25 | BV711 | 2A3 | BD | 563159 | Mouse BALB/c IgG1, κ | V13 |
| CCR4 | BV605 | L291H4 | Biolegend | 359418 | Mouse IgG1, κ | V10 |
| CCR6 | BV785 | G034 | Biolegend | 353422 | Mouse IgG2b, κ | V15 |

| | | | | | | |
|---|---|---|---|---|---|---|
| CXCR3 | PE | 1C6 | BD | 557185 | Mouse BALB/c IgG1, κ | YG1 |
| CXCR5 | PE-Cy7 | J252D4 | Biolegend | 356924 | Mouse IgG1, κ | YG9 |
| CCR10 | APC | 314305 | R&D | FAB3478A-100 | Rat IgG2A | R1 |
| CD69 | BUV661 | FN50 | BD | 750213 | Mouse IgG1, κ | UV11 |
| CD103 | PE/Fire700 | Ber-ACT8 | Biolegend | 350240 | Mouse IgG1, κ | YG7 |
| TCRγδ | PerCP-eFluor 710 | B1.1 | Thermo Fisher | 46-9959-42 | Mouse IgG1, κ | B10 |
| CD161 | PerCp-Cy5.5 | HP-3G10 | Biolegend | 339908 | Mouse IgG1, κ | B9 |

## Supplementary tables

### Supplementary Table-S1. Patient characteristics (overall cohort).

| Parameter | Baseline value |
|---|---|
| Age (years) | 59.2 (50.2-67.2) |
| Sex (M, %) | 163 (65) |
| Etiology (n, %)<br>- ALD<br>- Viral<br>- ALD + Viral<br>- NASH<br>- Cholestatic<br>- Other | <br>113 (45)<br>47 (19)<br>13 (5)<br>25 (10)<br>9 (4)<br>42 (17) |
| cACLD (n, %) | 110 (44) |
| EASL stage (n, %)<br>- S0 (HVPG 6-9 mmHg)<br>- S1-S2 (HVPG ≥10 mmHg without varices or presence of varices)<br>- S3 (Bleeding)<br>- S4 (Non-bleeding decompensation)<br>- S5 (Further decompensation) | <br>23 (9)<br>86 (34)<br>12 (5)<br>68 (27)<br>59 (24) |
| HVPG (mmHg) | 18 (12-21) |
| CTP stage<br>- A<br>- B<br>- C | <br>144 (58)<br>83 (33)<br>22 (9) |
| MELD score (points) | 11 (9-14) |
| Varices (n, %)<br>- None<br>- Small<br>- Large<br>- Unknown | <br>92 (37)<br>6 (26)<br>91 (37)<br>1 (0.4) |
| Ascites (n, %)<br>- None<br>- Mild/medically controlled<br>- Severe/refractory | <br>129 (52)<br>107 (43)<br>13 (5) |
| Hepatic encephalopathy (n, %)<br>- None<br>- Mild/medically controlled<br>- Severe | <br>198 (79)<br>51 (21)<br>0 (0) |
| Antibiotic prophylaxis (n, %)<br>- Rifaximin<br>- Norfloxacin<br>- Trimethoprim/Sulfamethoxazole | 20 (8)<br>15 (6)<br>4 (2)<br>1 (0.4) |
| HR (/min) | 76 (67-89) |
| MAP (mmHg) | 100 (91-111) |
| HR/MAP ratio | 0.75 (0.66-0.88) |
| Number of detectable bacterial antigens (n, %)<br>- None<br>- 1<br>- 2<br>- 3 | <br>9 (4)<br>61 (24)<br>119 (48)<br>60 (24) |
| LPS (EU/mL) | 0.64 (0.30-1.06) |

| | |
|---|---|
| LTA (pg/mL) | 43.2 (23.2-109) |
| bactDNA (yes; n, %) | 101 (41) |
| WBC (G/L) | 4.62 (3.30-5.94) |
| CRP (mg/dL) | 0.25 (0.11-0.53) |
| IL-6 (pg/mL) | 7.17 (4.28-12.0) |
| IL-10 (pg/mL) | 12.6 (9.40-16.2) |
| TNF-α (pg/mL) | 17.7 (11.3-30.6) |
| PCT (ng/mL) | 0.08 (0.05-0.13) |
| LBP (µg/mL) | 6.66 (5.08-8.59) |
| Copeptin (pmol/L) | 8.53 (4.32-15.9) |
| Renin (µIU/mL) | 29.5 (8.45-120) |

Abbreviations: (ALD) alcohol-related liver disease; (bactDNA) bacterial DNA; (CRP) C-reactive protein; (CTP) Child-Turcotte-Pugh; (EASL) European Association for the Study of the Liver; (HR) heart rate; (HVPG) hepatic venous pressure gradient; (IL-6/-10) interleukin-6/-10; (LBP) lipopolysaccharide binding protein; (M) male sex; (MAP) mean arterial pressure; (MELD) Model of End Stage Liver Disease; (NASH) non-alcoholic steatohepatitis; (PCT) procalcitonin; (TNF-α) tumor necrosis factor-alpha; (WBC) white blood cell

**Supplementary Table-S2. Baseline characteristics of patients with compensated and decompensated advanced chronic liver disease.**

| Parameter | Compensated ACLD (n=110) | Decompensated ACLD (n=139) | P-value |
|---|---|---|---|
| Age (years) | 59 (51-68) | 59 (50-66) | 0.709 |
| Sex (M, %) | 71 (65) | 92 (66) | 0.787 |
| Etiology (n, %)<br>- ALD<br>- Viral<br>- ALD + Viral<br>- NASH<br>- Cholestatic<br>- Other | <br>26 (24)<br>35 (32)<br>5 (5)<br>21 (19)<br>5 (5)<br>18 (16) | <br>87 (63)<br>12 (9)<br>8 (6)<br>4 (3)<br>4 (3)<br>24 (17) | **<0.001** |
| HVPG (mmHg) | 13 (9-18) | 19 (16-23) | **<0.001** |
| MELD Score (points) | 9 (8-12) | 13 (10-16) | **<0.001** |
| HR (bpm) | 77 (68-86) | 75 (67-90) | 0.930 |
| MAP (mmHg) | 106 (97-113) | 98 (88-107) | **<0.001** |
| HR/MAP ratio | 0.73 (0.62-0.86) | 0.78 (0.67-0.91) | **0.018** |
| Detectable bacterial antigens (n, %)<br>- None<br>- 1<br>- 2<br>- 3 | <br><br>2 (2)<br>30 (27)<br>55 (50)<br>23 (21) | <br><br>7 (5)<br>31 (22)<br>64 (46)<br>37 (27) | 0.333 |
| LPS (EU/mL) | 0.89 (0.41-1.26) | 0.68 (0.31-1.29) | 0.505 |
| LTA (pg/mL) | 41.3 (26.3-107) | 37.5 (21.4-133) | 0.653 |
| BactDNA (n, %) | 46 (42) | 55 (40) | 0.720 |
| WBC (G/L) | 4.81 (3.49-6.22) | 4.44 (3.17-5.74) | **0.039** |
| CRP (mg/dL) | 0.19 (0.08-0.35) | 0.32 (0.14-0.66) | **<0.001** |
| IL-6 (pg/mL) | 5.34 (3.24-8.53) | 8.83 (5.39-15.3) | **<0.001** |
| IL-10 (pg/mL) | 12.5 (8.90-16.3) | 13.3 (10.4-16.4) | 0.070 |
| TNF-α (pg/mL) | 18.4 (10.6-31.4) | 15.8 (8.93-37.4) | **0.558** |
| TNF-α/IL-10 ratio | 1.61 (1.08-2.22) | 1.34 (0.76-2.20) | **0.071** |
| Procalcitonin (ng/mL) | 0.07 (0.04-0.10) | 0.09 (0.05-0.15) | **<0.001** |
| LBP (µg/mL) | 6.63 (5.43-8.63) | 6.67 (4.88-8.33) | 0.429 |
| Copeptin (pmol/L) | 6.07 (3.50-14.1) | 9.49 (5.14-16.6) | **0.041** |
| Renin (µIU/mL) | 12.9 (5.03-29.5) | 57.4 (21.6-233) | **<0.001** |

Abbreviations: (ALD) alcohol-related liver disease; (bactDNA) bacterial DNA; (CRP) C-reactive protein; (HR) heart rate; (HVPG) hepatic venous pressure gradient; (IL-6/-10) interleukin-6/-10; (LBP) lipopolysaccharide binding

protein; (M) male sex; (MAP) mean arterial pressure; (MELD) Model of End Stage Liver Disease; (NASH) non-alcoholic steatohepatitis; (PCT) procalcitonin; (TNF-α) tumor necrosis factor-alpha; (WBC) white blood cell

## Supplementary figures

*Patient flow chart*

**Supplementary Figure-S1. Patient flow chart.**

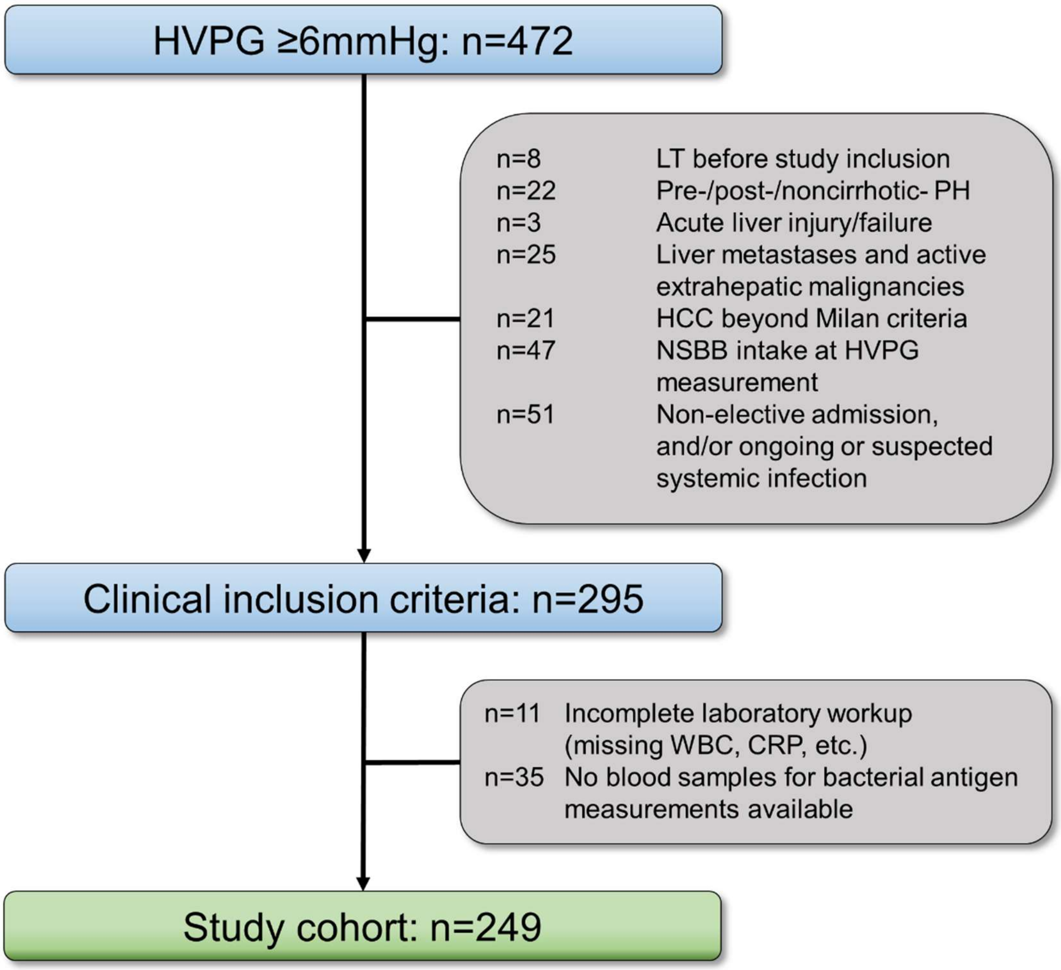


Abbreviations: (LT) liver transplantation; (PH) portal hypertension; (HCC) hepatocellular carcinoma; (NSBB) non-selective betablocker; (HVPG) hepatic venous pressure gradient; (WBC) white blood cell; (CRP) C-reactive protein

*Relationship between different bacterial translocation markers*

**Supplementary Figure-S2. Correlation between different bacterial antigens in the systemic circulation.**

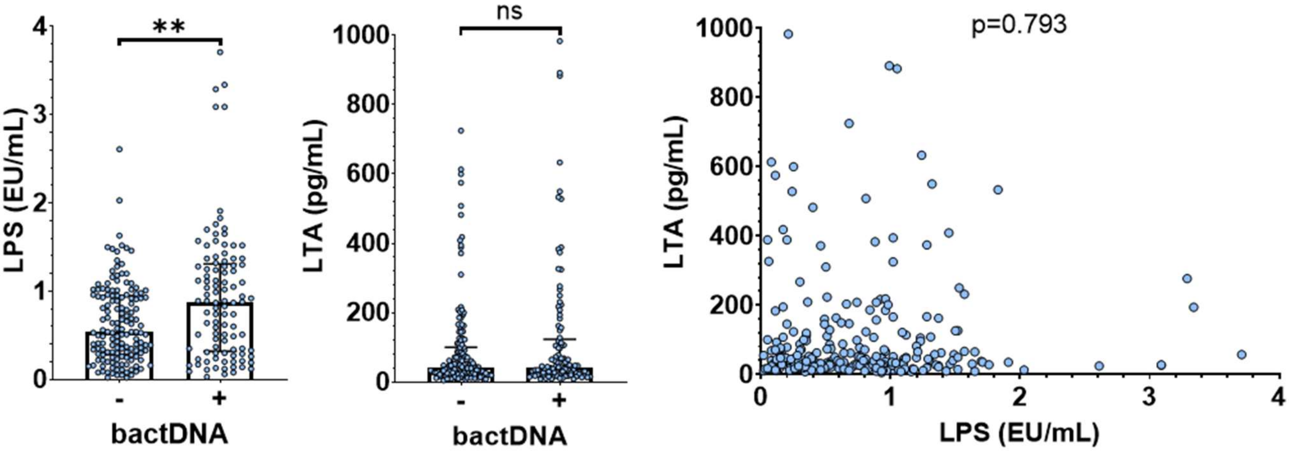


*Bacterial translocation markers and disease stage, ascites, or etiology*

**Supplementary Figure-S3. Bacterial antigen levels in patients stratified by the presence and severity of ascites.**

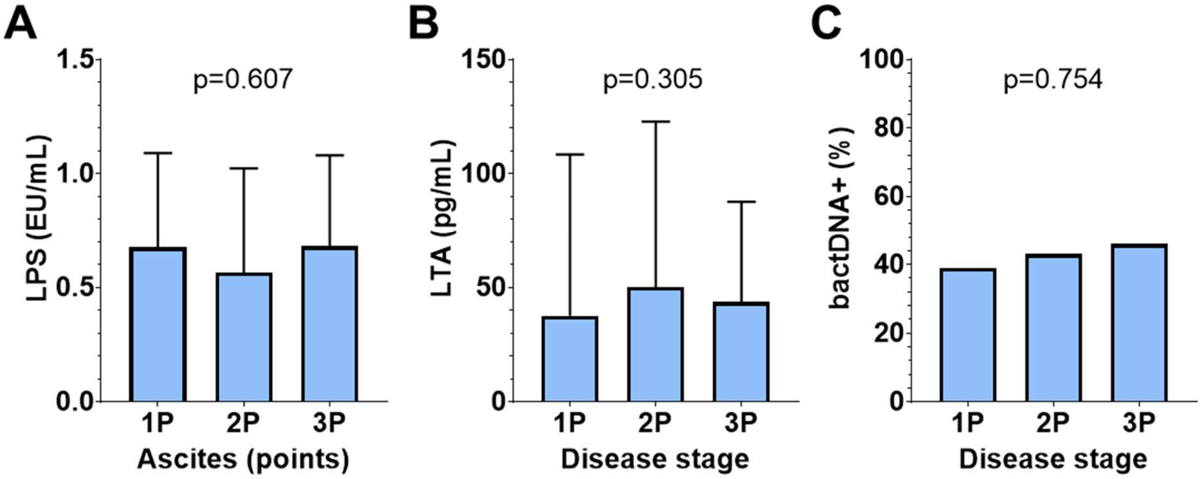


Legend: Ascites grading: 1 = no ascites (n=129); 2 = mild/medically controlled ascites (n=107); 3 = severe/refractory ascites (n=13). Statistical analysis: Continous variables were compared by Kruskal-Wallis test. Categorical variables were compared by Chi-squared test. Abbreviations: (LPS) lipopolysaccharide; (LTA) lipoteichoic acid; (bactDNA) bacterial DNA

**Supplementary Figure-S4. Bacterial antigen levels in patients stratified by etiology of liver disease.**

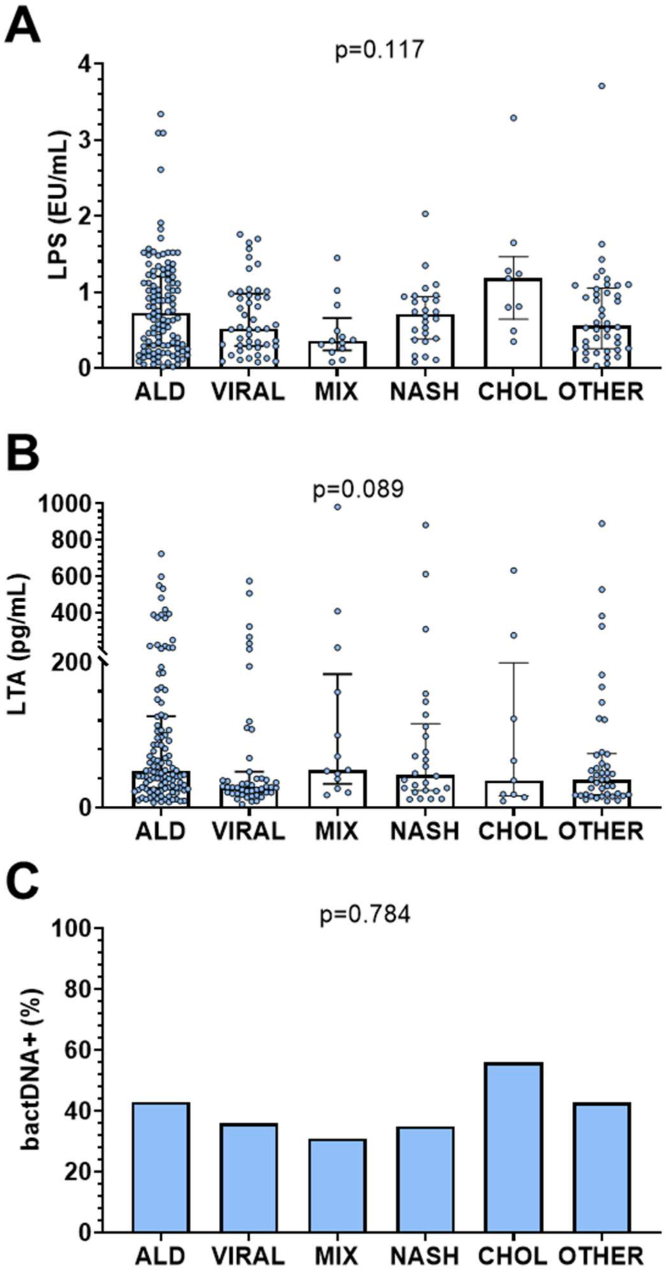


Statistical analysis: Continous variables were compared by Kruskal-Wallis test. Categorical variables were compared by Chi-squared test. Abbreviations: (LPS) lipopolysaccharide; (LTA) lipoteichoic acid; (bactDNA) bacterial DNA

**Supplementary Figure-S5. Bacterial antigen levels in the study cohort after exclusion of patients on antibiotic prophylaxis.**

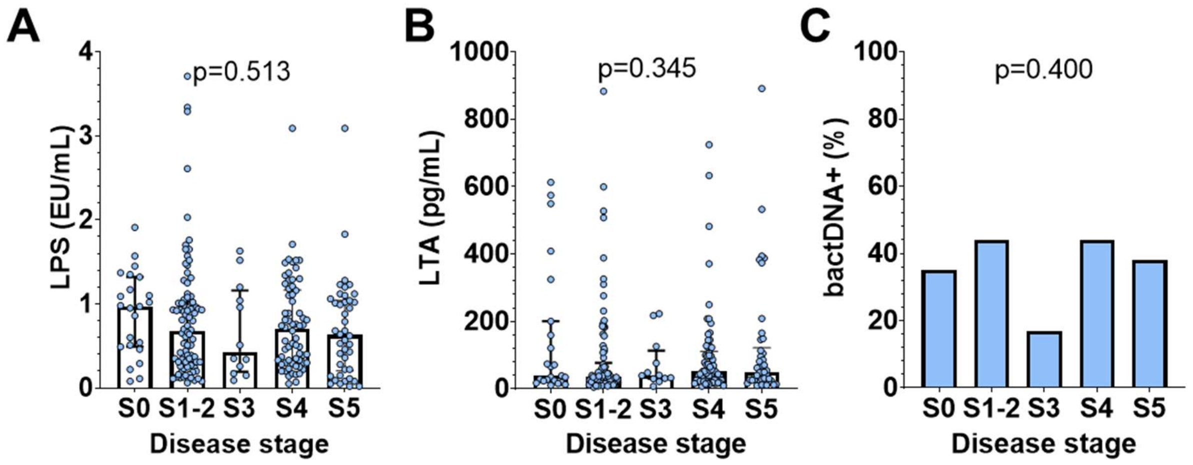


Statistical analysis: Continous variables were compared by Kruskal-Wallis test. Categorical variables were compared by Chi-squared test. Abbreviations: (LPS) lipopolysaccharide; (LTA) lipoteichoic acid; (bactDNA) bacterial DNA; (S0-S5) clinical disease stages

*Bacterial translocation markers and systemic inflammation*

**Supplementary Figure-S6. Correlation between bacterial antigens, CRP, IL-6, and TNF-α in the systemic circulation.**

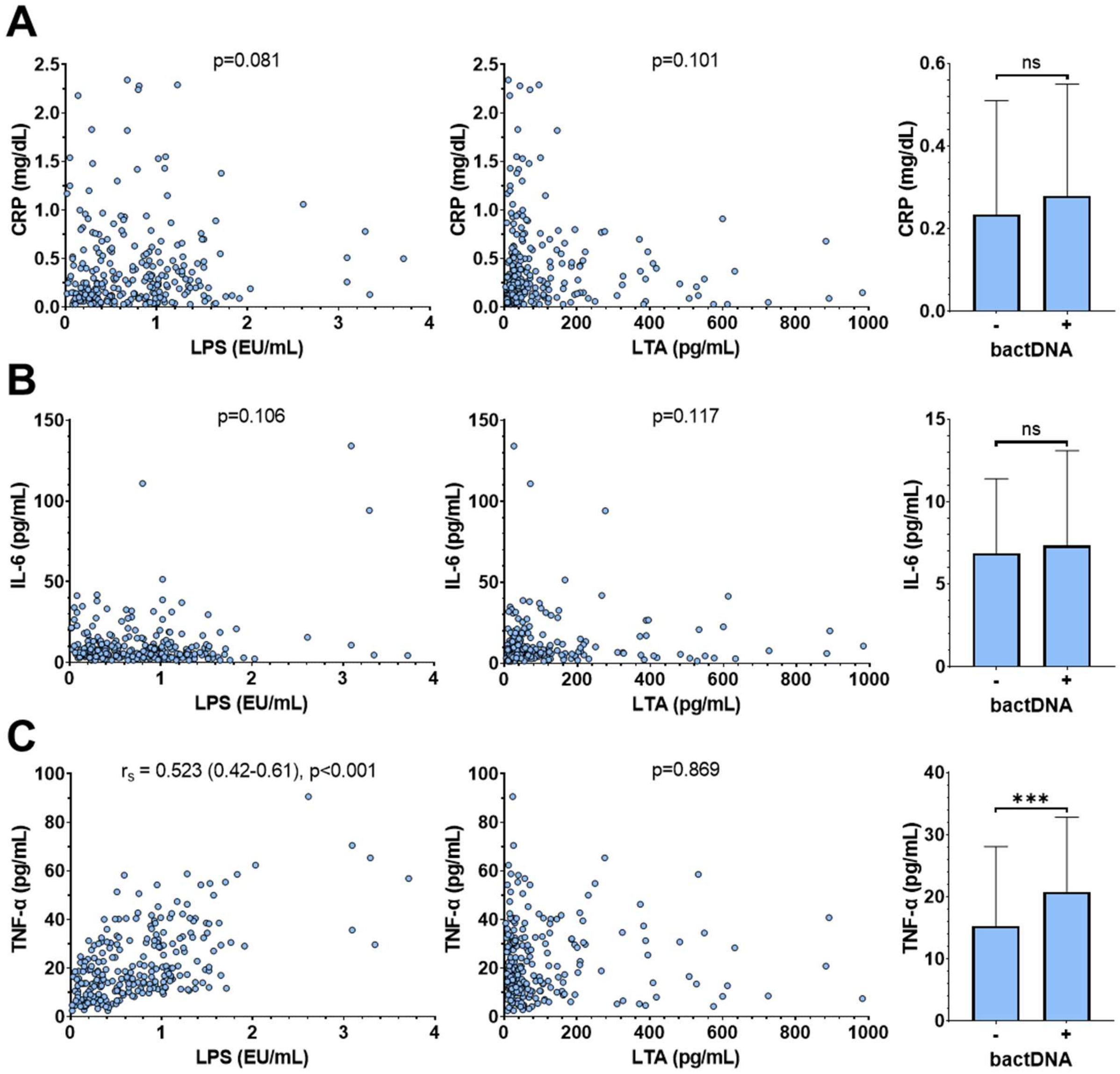


Statistical analysis: Spearman's correlation coefficients were calculated to assess the association between continuous variables. Mann-Whitney U test was applied to compare continuous variables. Legend: (ns) not significant, (*) p<0.05, (**) p<0.01, (***) p<0.001. Abbreviations: (LTA) lipoteichoic acid; (LPS) lipopolysaccharide; (bactDNA) bacterial DNA; (CRP) C-reactive protein; (IL-6) interleukin-6); (LBP) LPS-binding protein

**Supplementary Figure-S7. Correlation between bacterial antigens and inflammation markers in the systemic circulation.**

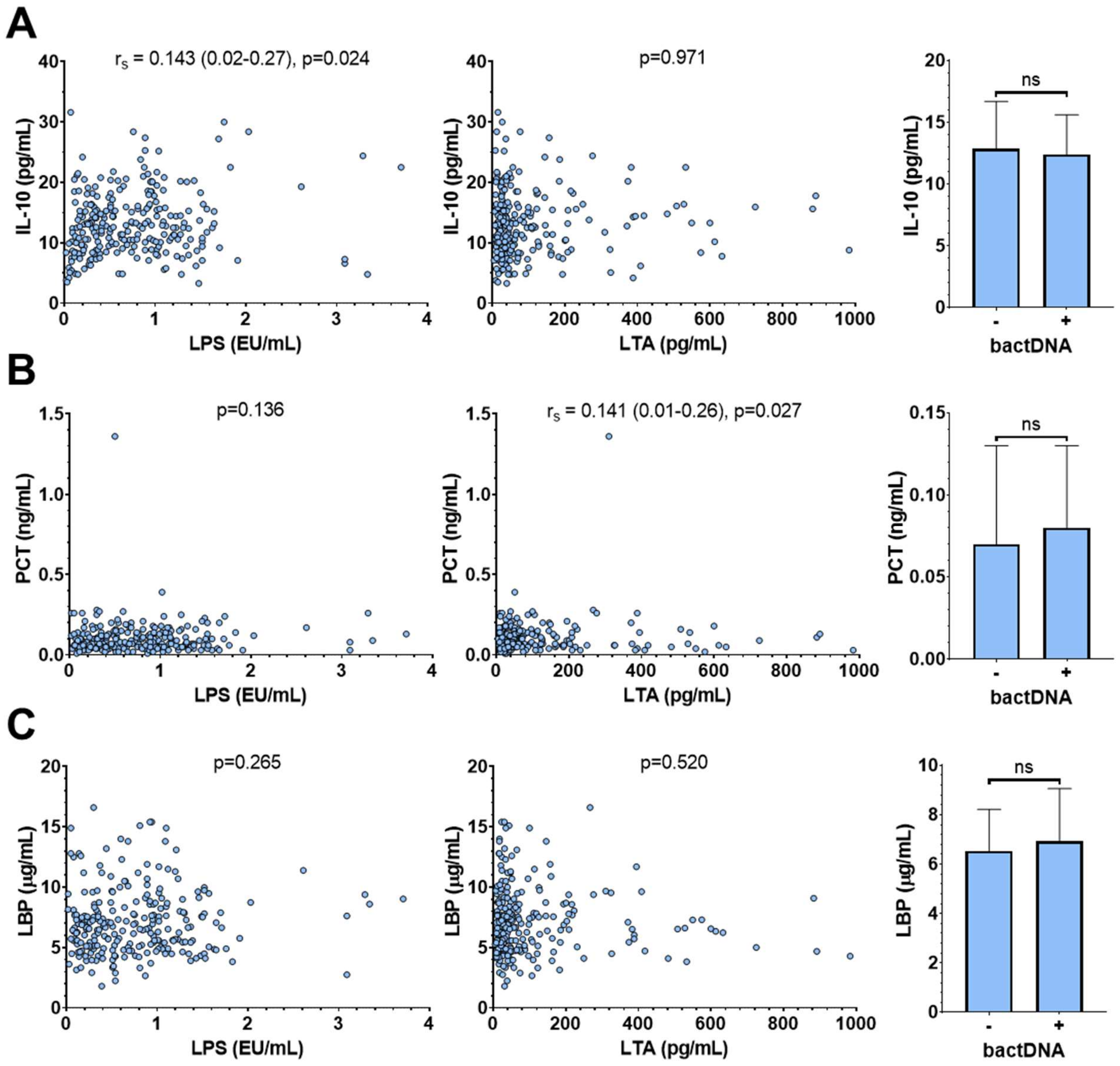


Statistical analysis: Spearman's correlation coefficients were calculated to assess the association between continuous variables. Mann-Whitney U test was applied to compare continuous variables. Legend: (ns) not significant, (*) p<0.05, (**) p<0.01, (***) p<0.001. Abbreviations: (LTA) lipoteichoic acid; (LPS) lipopolysaccharide; (bactDNA) bacterial DNA; (CRP) C-reactive protein; (IL-6) interleukin-6); (LBP) LPS-binding protein

**Supplementary Figure-S8. Correlation between bacterial antigens and measures of hepatic and systemic hemodynamics.**

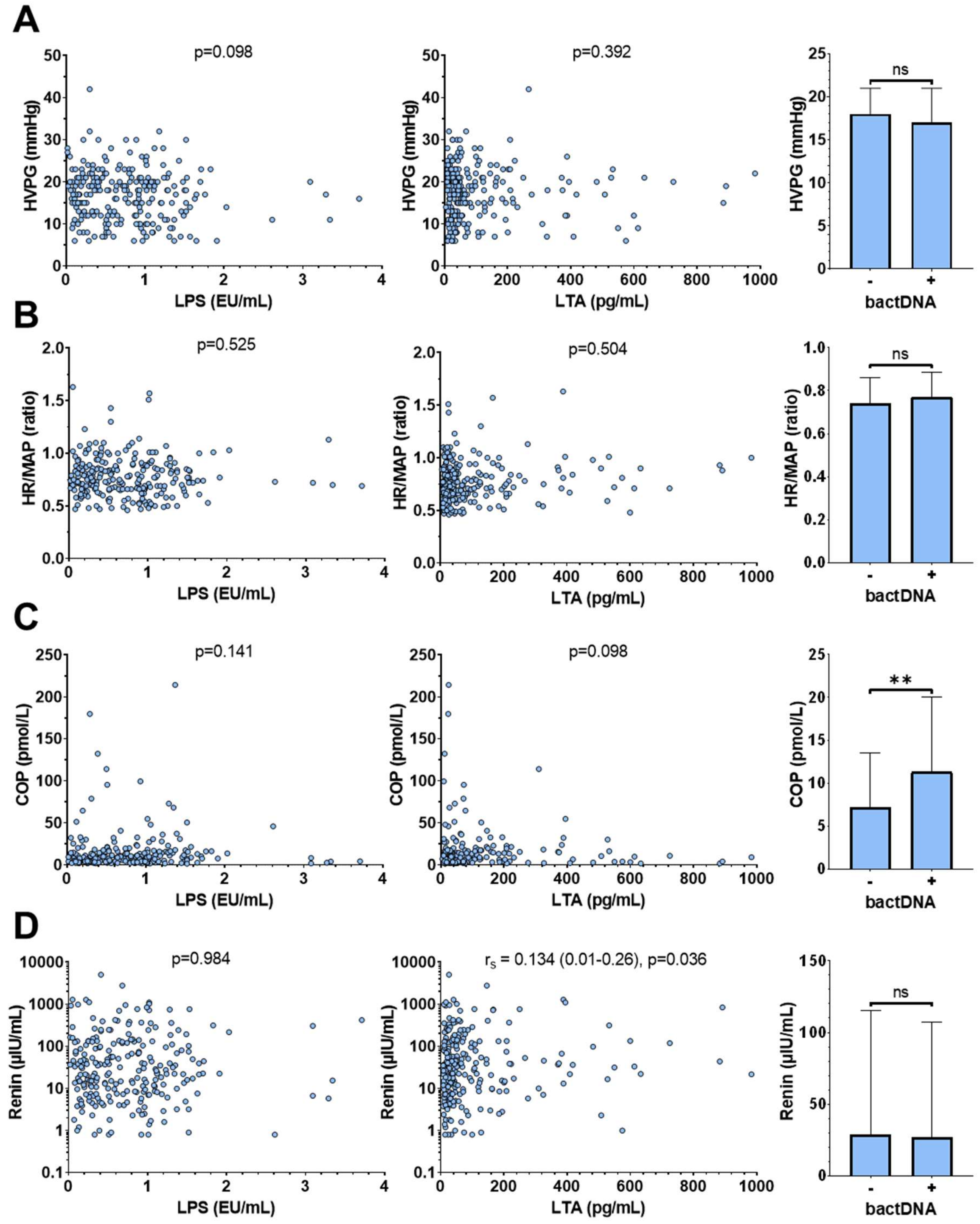


Statistical analysis: Spearman's correlation coefficients were calculated to assess the association between continuous variables. Mann-Whitney U test was applied to compare continuous variables. Legend: (ns) not significant, (*) p<0.05, (**) p<0.01, (***) p<0.001. Abbreviations: (LTA) lipoteichoic acid; (LPS) lipopolysaccharide; (bactDNA) bacterial DNA; (CRP) C-reactive protein; (IL-6) interleukin-6); (LBP) LPS-binding protein; (HVPG) hepatic venous pressure gradient; (COP) copeptin

**Supplementary Figure-S9. Incidence of first/further decompensation or liver-related death in patients stratified by median LPS/LTA levels and the presence of bactDNA in the systemic circulation.**

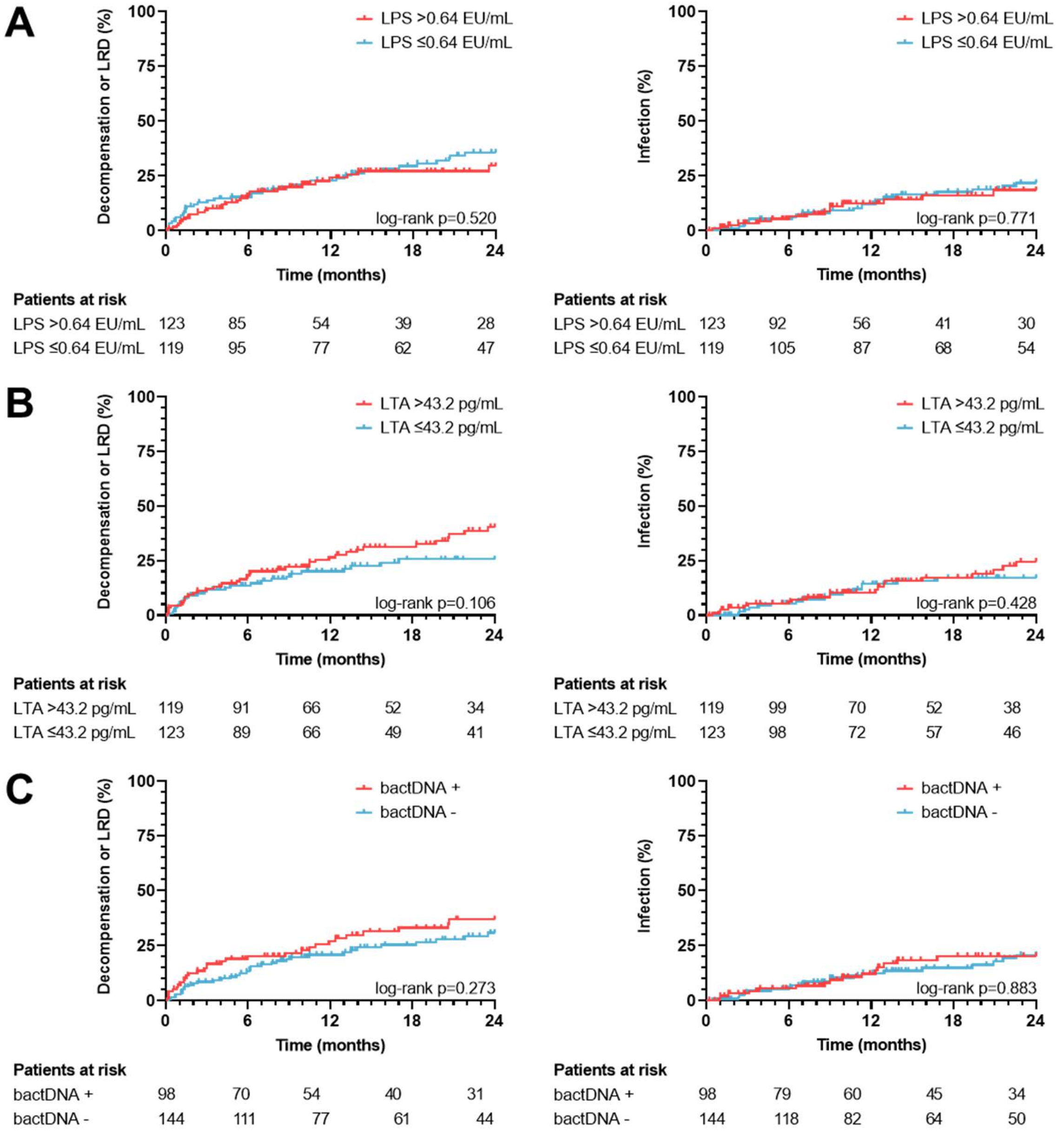


Statistical analysis: The incidence of events in different patient groups was compared by log-rank test.

Abbreviations: (LTA) lipoteichoic acid; (LPS) lipopolysaccharide; (bactDNA) bacterial DNA

**Supplementary Figure-S10. Gating strategy for T-cell subsets in intestinal mucosa biopsies.**

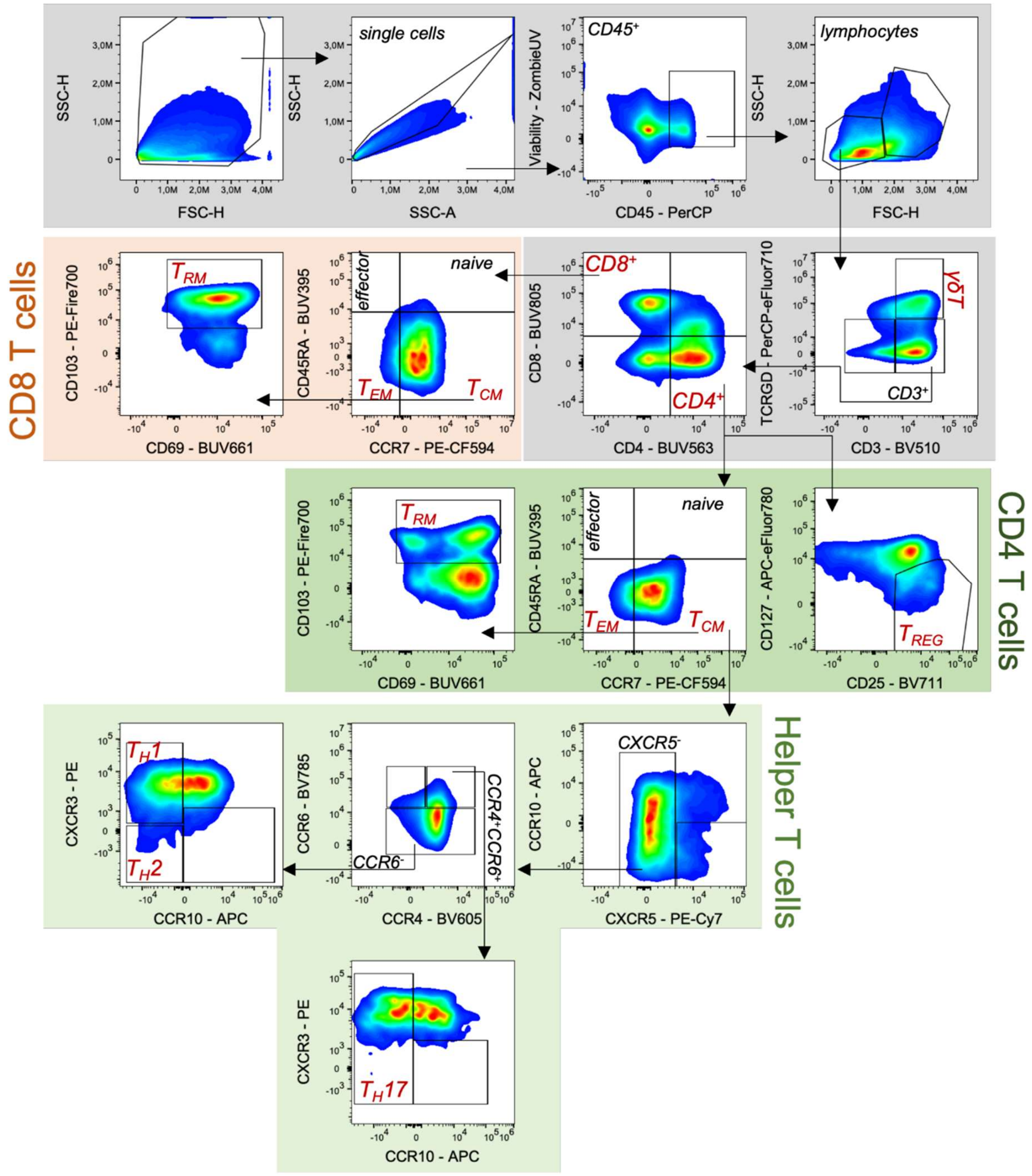

## References (used in the supplementary material)

1. Reiberger, T., et al., *Measurement of the Hepatic Venous Pressure Gradient and Transjugular Liver Biopsy.* J Vis Exp, 2020(160).
2. Roth, R.I., F.C. Levin, and J. Levin, *Optimization of detection of bacterial endotoxin in plasma with the Limulus test.* J Lab Clin Med, 1990. **116**(2): p. 153-61.
